# Comparison of Compressive and Flexural Strength Among Three Types of Glass Ionomer Cements: An In Vitro Study

**DOI:** 10.7759/cureus.87171

**Published:** 2025-07-02

**Authors:** Mayank Chaudhary, Amit Kumar, Prasanta Majumder, Rajnish Kumar, Ankit Kumar Saha, Paramarshi Das

**Affiliations:** 1 Department of Conservative Dentistry and Endodontics, Mithila Minority Dental College and Hospital, Darbhanga, IND; 2 Department of Public Health Dentistry, Agartala Government Dental College and Indira Gandhi Memorial (IGM) Hospital, Agartala, IND; 3 Department of Conservative Dentistry and Endodontics, Ex-Servicemen Contributory Health Scheme (ECHS) Polyclinic, Agartala, IND

**Keywords:** compressive strength, glass ionomer cement, resin-modified glass ionomer cement, restorative dental material, restorative dentistry

## Abstract

Background

Glass ionomer cements (GICs) are widely used restorative materials in dental procedures as they release fluorides, chemically adhere to tooth structure, and are biocompatible. Despite these advantages, their mechanical properties vary. Hence, this study aimed to compare the compressive and flexural strengths of three types of GICs, namely, conventional GIC, resin-modified GIC (RMGIC), and zirconia-reinforced GIC (Zirconomer).

Methodology

An in vitro study was conducted on 120 extracted natural teeth. The 120 specimens were divided into the following three groups of 40 samples: Group A (RMGIC; GC FujiCem; 13.3 g), Group B (conventional GIC; GC Gold Label 2; 15 g powder, 10 mL liquid), and Group C (Zirconomer; SHOFU Zirconomer Improved; 12 g powder, 5 mL liquid). Plastic straws measuring 4 mm in diameter and 6 mm in height were used as molds. Materials were manipulated according to the manufacturer’s instructions, with light curing performed for RMGIC. All samples were coated with GIC varnish and stored in deionized water at 37°C for 30 days. Flexural strength was tested using rectangular specimens (4 × 6 × 25 mm) on a Universal Testing Machine after 24 hours of water storage. Compressive strength was measured on cylindrical specimens using an INSTRON E 3000 universal testing machine at a crosshead speed of 1 mm/minute, and values were calculated using the following formula: CS = 4F/πD². Statistical analysis was performed using analysis of variance and Bonferroni post-hoc tests.

Results

Statistically significant mean measures were seen among all three groups. Group B (conventional GIC) showed the highest compressive and flexural strength, which was statistically significant, compared to the other two groups.

Conclusions

Within the limits of the study, conventional GIC had higher compressive strength and flexural strength, indicating enhanced durability.

## Introduction

Historically, gold and ceramics used to be the material of choice for indirect restorations, while dental amalgam was predominantly used for direct restorations. Over time, the use of amalgam as a restorative material declined due to concerns over its mercury-related toxicity and risk of allergic reactions [1,2]. In response to these concerns, the Minamata Convention on Mercury was initiated to phase down the use of dental fillings containing mercury [3]. With scientific advancements, various other materials for direct restorations were developed, such as glass ionomer cement (GIC), composite resins, and compomers. Of these, GICs are widely used in restorative dentistry owing to their unique properties, including chemical adhesion to tooth structure, fluoride release, and biocompatibility [4,5]. GICs are used in various clinical applications because their physical characteristics can be altered by adjusting the powder-to-liquid ratio or modifying their chemical composition. Moreover, the inclusion of fluoride imparts anti-cariogenic properties, along with favorable biocompatibility and strong chemical bonding to mineralized tissues [6,7]. However, notable shortcomings such as low tensile, compressive, and flexural strength have been observed in conventional GICs [8]. Over time, GICs have undergone advancements, including modifications in their composition [9]. To strengthen the mechanical properties, resin-modified glass ionomers were introduced to increase the material’s strength [10]. The binding strength, tensile strength, and compressive strength of GICs are maintained as a result of resin addition, while their solubility in oral environments is reduced, thus correcting GIC limitations [11]. Zirconia-reinforced GIC (Zirconomer and Zirconomer enhanced) is another modern variant of GIC introduced by SHOFU that includes zirconia, a highly durable ceramic [12]. The compressive strength of GICs ranges from 60 to 300 MPa, while the flexural strength is recorded to be around 50 MPa. According to previous reports, GIC exhibits an increase in flexural and comprehensive strength when exposed to water between 24 hours to one year after mixing [13,14]. These mechanical properties are crucial to influence the longevity and success of the restorative procedures. With the increasing variety of GICs available, it is essential to evaluate and compare the mechanical properties systematically. While numerous studies have individually investigated these materials, in vitro comparisons are still limited [15,16]. Therefore, this study aimed to compare the compressive and flexural strength of three different types of commercially available GICs, namely, conventional GIC (GC Gold Label 2), resin-modified GIC (RMGIC), and zirconia-reinforced GIC (Zirconomer). The results of this study will aid clinicians in improving clinical outcomes.

## Materials and methods

Study design and setting

An in vitro study was conducted from January 2024 to March 2025 in the Department of Conservative Dentistry and Endodontics at Mithila Minority Dental College and Hospital in Bihar, India. Ethical approval was obtained from the Institutional Ethical Committee (approval number: EC/NEW/INST/2023/4152/Ref 07). The study complies with the Checklist for Reporting In Vitro Studies (CRIS) norms and the Declaration of Helsinki principles.

Sample size estimation

The sample size for this investigation was estimated using G*Power software version 3.6.9 (Heinrich Heine University Düsseldorf, Düsseldorf, Germany), with a power of 80% and an alpha error of 5%. To examine the mean difference in flexural and compressive strength across the research groups, an effect size of 0.56 was calculated using data from a reference study [17]. Based on these criteria, the projected sample size was calculated to be 120, with 40 samples of natural teeth in each study group.

Specimen preparation and group allocation

**Figure 1:**
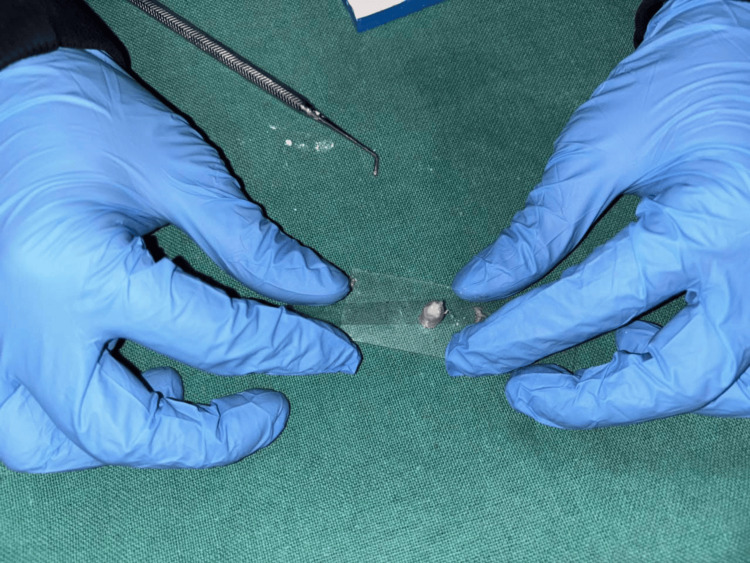
Specimen preparation.

A total of 120 cylindrical specimens were prepared for the study using plastic straws (4 mm diameter × 6 mm height) as molds. To prevent adhesion, the inner surfaces of the straws were coated with petroleum jelly. Each GIC type was mixed in accordance with the manufacturer’s guidelines. The material was introduced into the molds, which were positioned on a glass slide, covered with a Mylar strip, and pressed with another glass slide to achieve a smooth, even surface (Figure 1).

The extracted human premolar teeth specimens were randomly divided into the following three groups based on the type of GIC used: Group A = RMGIC (GC FujiCem; 13.3 g), Group B = conventional GIC (GC Gold Label 2; 15 g powder, 10 mL liquid), and Group C = Zirconomer (Zirconomer improved; 12 g powder, 5 mL liquid).

**Figure 2:**
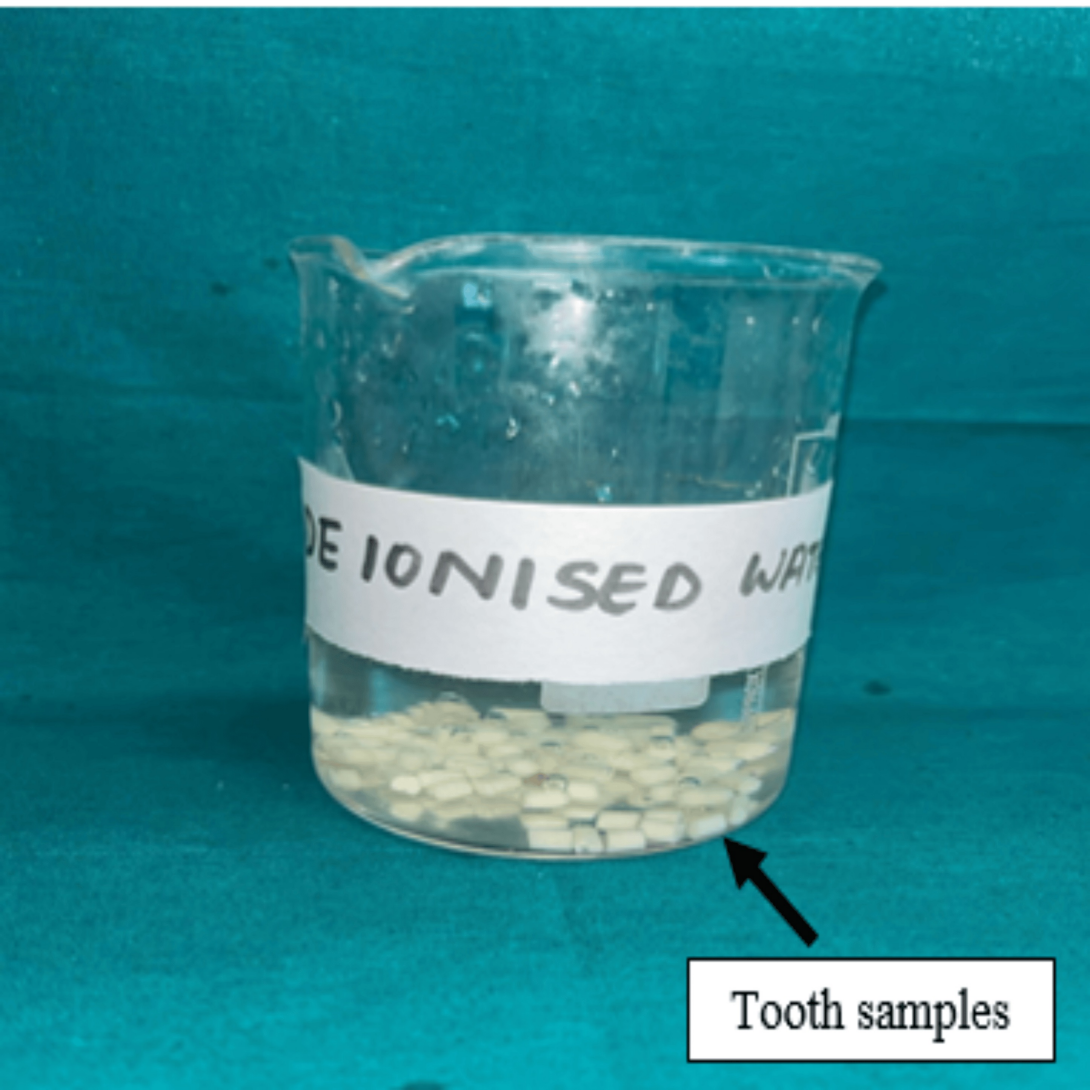
Specimens immersed in de-ionized water. The natural tooth specimens were immersed in de-ionized water at 37°C.

For Group A (n = 40), the RMGIC specimens were light-cured from all sides for 20 seconds using an LED curing unit (Woodpecker LED D Curing Light; wavelength: 420-480 nm; intensity: 1,000-1,700 mW/cm²), maintaining a standardized distance of 1-2 mm. The samples in Groups B and C were allowed to set undisturbed for the time specified by their respective manufacturers. Once set, specimens were demolded and polished with #600 silicon carbide paper to eliminate surface irregularities. A protective GIC varnish was applied to each sample to guard against moisture exposure and left undisturbed for two minutes for solvent evaporation [18]. All specimens were then stored in deionized water at 37°C for 30 days to simulate intraoral conditions (Figure 2), with weekly replacement of the water to maintain sample stability [19].

Methodology

Testing Equipment

The INSTRON E 3000 universal testing machine was employed for measuring flexural and compressive strength.

Flexural Strength Testing

Specimens (4 × 6 × 25 mm) were prepared in stainless steel molds, cured or set, and finished with abrasive paper. RMGIC was light-cured for 20 seconds. Samples were stored in deionized water at 37°C for 24 hours, and then tested for flexural strength using the INSTRON E 3000 universal testing machine.

Compressive Strength Testing

Cylindrical specimens (4 × 6 mm) were prepared in aluminum molds using materials mixed per the manufacturer’s instructions. After setting in a moist environment for 10 minutes, RMGIC samples were light-cured for 20 seconds on each side. Testing was performed at 23 ± 1°C using the INSTRON E 3000 universal testing machine at 1 mm/minute. Compressive strength was calculated using the following formula: CS = 4F/πD², where F is the maximum load and D is the 4 mm diameter.

Statistical analysis

The collected data were methodically entered into Microsoft Excel (Microsoft Corp., Redmond, WA, USA) for further inspection and then analyzed using SPSS version 21.0 software (IBM Corp., Armonk, NY, USA). The Shapiro-Wilk test was used to assess the normality of the data distribution, and it was determined that the data were normal. Continuous variables are shown as means and standard deviations (SDs). To assess statistically significant differences among the outcome variables, one-way analysis of variance was performed on the flexural and compressive strength scores, followed by the Bonferroni post-hoc test. The statistical analyses were performed at a confidence interval (CI) of 95%. The p-value was fixed at 0.05.

## Results

A Cohen’s kappa coefficient of 0.88 indicated excellent inter-rater reliability and reproducibility in the current study. The findings revealed statistically significant differences in both parameters, i.e., compressive strength and flexural strength, among the three groups. Group B (conventional GIC) had the highest compressive strength (231.7 ± 4.3 MPa), followed by Group C (193.2 ± 5.0 MPa) and Group A (162.9 ± 8.4 MPa) (Table 1). Similarly, for flexural strength, Group B achieved the highest value (80.6 ± 2.0), while Group C and Group A exhibited lower strengths (Table 2).

**Table 1:** Comparison of the mean compressive strength among three types of glass ionomer cements using one-way analysis of variance. *: P-value <0.05 is considered statistically significant. Group A = resin-modified glass ionomer cements (GC FujiCem; 13.3 g); Group B = conventional glass ionomer cements (GC Gold Label 2; 15 g powder, 10 mL liquid); and Group C = Zirconomer (Zirconomer Improved; 12 g powder, 5 mL liquid). The data are presented as the mean and standard deviation.

Group	Mean	Standard deviation	F-value	P-value
Group A	162.9 MPa	8.4	619.6	0.00*
Group B	231.7 MPa	4.3		
Group C	193.2 MPa	5.0		

**Table 2:** Comparison of the mean flexural strength among three types of glass ionomer cements using one-way analysis of variance. *: P-value <0.05 is considered statistically significant. Group A = resin-modified glass ionomer cements (GC FujiCem; 13.3 g); Group B = conventional glass ionomer cements (GC Gold Label 2; 15 g powder, 10 mL liquid); and Group C = Zirconomer (Zirconomer Improved; 12 g powder, 5 mL liquid). The data are presented as the mean and standard deviation.

Group	Mean	Standard deviation	F-value	P-value
Group A	69.9 MPa	5.8	44.5	0.00*
Group B	80.6 MPa	2.0		
Group C	73.8 MPa	1.2		

## Discussion

This study aimed to compare the compressive and flexural strength of three different types of GICs. The results revealed statistically significant differences among the groups, with Group B (conventional GIC) demonstrating superior mechanical properties in both compressive and flexural strength compared to Groups A and C. The findings underscore the effect of composition on the mechanical performance of a restorative material.

GICs are one of the most widely used restorative materials, especially in minimally invasive dentistry. As they are intended to replace lost tooth structure, they must possess sufficient mechanical strength to endure functional forces such as mastication. Among various mechanical tests, compressive strength is commonly used to assess the structural resilience of these materials, as it serves as a reliable indicator of their capacity to withstand occlusal forces [20].

The ISO 4049:2019 mandates the compressive strength of GICs to be within 100-200 MPa [21]. In the current study, the compressive strength was found to be the highest, in line with the mandate by ISO 4049:2019, in conventional GICs, which may be attributed to increased silica content and finer particle size. Similar findings have been reported by other studies, where conventional GICs outperformed RMGICs under conditions of storage in water medium [22,23].

GICs are the choice of restoration in dental cavities and facial surfaces involving cervical portions of the tooth. In such cases, materials with a high modulus of elasticity may not effectively accommodate the changes in tooth form caused due to flexural forces, leading to an increased risk of fracture of the restoration or marginal breakdown. Flexural strength becomes an important aspect of a restorative material in this context, as it reflects a material’s ability to resist deformation and fracture under bending forces, directly influencing the outcome of a restoration [24]. Flexural strength of conventional GICs was better compared to RMGICs and Zirconomer in this study. Contradicting results have been reported in other studies where conventional GICs performed poorly in terms of flexural strength [25,26]. This discrepancy may be attributed to variations in curing or specimen maturation time. The strength of conventional GICs develops gradually through an acid-base reaction, whereas RMGICs and Zirconomer rely on a hybrid setting mechanism [27].

While conventional GICs demonstrated superior compressive and flexural strength compared to RMGICs and Zirconomer in this study, these findings should be interpreted along with factors such as material choice, storage conditions, and testing methods. Additionally, advanced and analytical techniques such as micro-CT could provide deeper insights into filler homogeneity and matrix integrity. The study correspondingly underscores the necessity of conducting strength tests after a delay of 24 hours or more to achieve precise measurements of GIC strength. Furthermore, these findings may influence the development of future GIC formulations, suggesting that simpler conventional formulations might sometimes outperform more complex modified versions for specific mechanical properties.

Clinical implications

While RMGICs and Zirconomer are often selected for their improved mechanical properties, this study suggests that conventional GICs may still be preferable in certain clinical situations requiring optimal compressive and flexural strength, such as core build-ups and Class V restorations. The result emphasizes the importance of proper material handling and manipulation techniques, as these could significantly affect the performance of the restorative material. A balanced approach, considering both evidence-based research and clinical experience, remains essential for ideal restorative procedures.

Study limitations

This study has certain limitations to be considered. Due to the in vitro nature of the study, the findings may not fully replicate clinical conditions where factors such as moisture and temperature affect a restoration. The mechanical properties were assessed immediately or after a limited storage period, whereas long-term aging and fatigue resistance in clinical settings remain unknown.

## Conclusions

This study highlights significant differences in compressive and flexural strengths among three GIC types, with conventional GICs showing superior properties. The findings align with existing literature, emphasizing the impact of material composition on mechanical performance. These results are critical for restorative and prosthetic dentistry, where stronger materials enhance durability and resistance to occlusal forces. Future research should explore long-term clinical performance, wear resistance, bonding ability, and biocompatibility. Advancements in dental material science can lead to the development of optimized, durable, and biocompatible materials, ultimately improving the longevity and success of dental restorations and prostheses in clinical settings.
